# Percutaneous stenting for late outflow graft obstruction in left ventricular assist device: A case report

**DOI:** 10.1016/j.jvscit.2026.102423

**Published:** 2026-07-24

**Authors:** Mohammad E. Barbati, Shirin S. Ghiasi, Mohamed Elkady, Christoph S. Mueller, Nikolaos Tsilimparis, Jan Stana

**Affiliations:** a Department of Vascular Surgery, LMU University Hospital, LMU Medizin, Ludwig-Maximilians-Universität München, Munich, Germany; b Department of Cardiology and Angiology, Hannover Medical School, Hanover, Germany; c Department of Vascular Surgery, Faculty of Medicine, Assiut University, Assiut, Egypt; d Department of Cardiac Surgery, LMU University Hospital, LMU Medizin, Ludwig-Maximilians-Universität München, Munich, Germany

**Keywords:** HeartMate 3, Left ventricular assist device, Outflow graft obstruction, Percutaneous endovascular stenting, Self-expanding venous stent

## Abstract

Left ventricular assist device (LVAD) outflow graft obstruction (OGO) is an uncommon but serious late complication requiring individualized treatment. We describe a 71-year-old HeartMate 3 recipient with long-segment OGO stenosis more than 3 years after implantation. Because surgical revision was high risk, overlapping self-expanding venous stents were implanted percutaneously, with brief LVAD deactivation enabling controlled proximal deployment near the pump. Imaging confirmed restored graft patency and improved flow. A later distal thrombotic stenosis was managed conservatively with intensified anticoagulation. Percutaneous stenting with tailored procedural safeguards can safely treat complex late LVAD OGO in selected patients.

Continuous flow left ventricular assist devices (LVADs) are well established for advanced heart failure, serving as a bridge to transplantation or destination therapy. Improvements in pump design and anticoagulation strategies have significantly reduced the incidence of pump thrombosis, particularly with newer fully magnetically levitated systems such as the HeartMate 3 (Abbott Laboratories).^1^, ^2^, ^3^ Nevertheless, late complications involving the LVAD outflow graft have emerged as an increasingly recognized cause of device dysfunction.

Late outflow graft obstruction (OGO), typically occurring months to years after LVAD implantation, may present with low-flow alarms, hemodynamic compromise, or incidentally on imaging.^3^ Notably, laboratory parameters, such as hemolysis markers and LVAD log-file trends, may remain stable, potentially delaying diagnosis.^1^, ^2^, ^3^, ^4^ Computed tomography angiography (CTA) has therefore become central to diagnostic evaluation and procedural planning, allowing precise assessment of lesion location, extent, and severity.^3^^,^^4^

Management options for OGO include surgical revision, heart transplantation, percutaneous endovascular stenting, or conservative anticoagulation strategies. However, evidence guiding the optimal approach remains limited, and decision-making is often individualized, particularly in patients with high surgical risk or complex anatomy.^2^^,^^3^^,^^5^, ^6^, ^7^, ^8^, ^9^, ^10^ Percutaneous stenting has emerged as an effective alternative when surgery is contraindicated, yet concerns remain regarding procedural risks, distal migration of obstructive material, and postintervention thrombosis (Fig 1).

**Fig 1:**
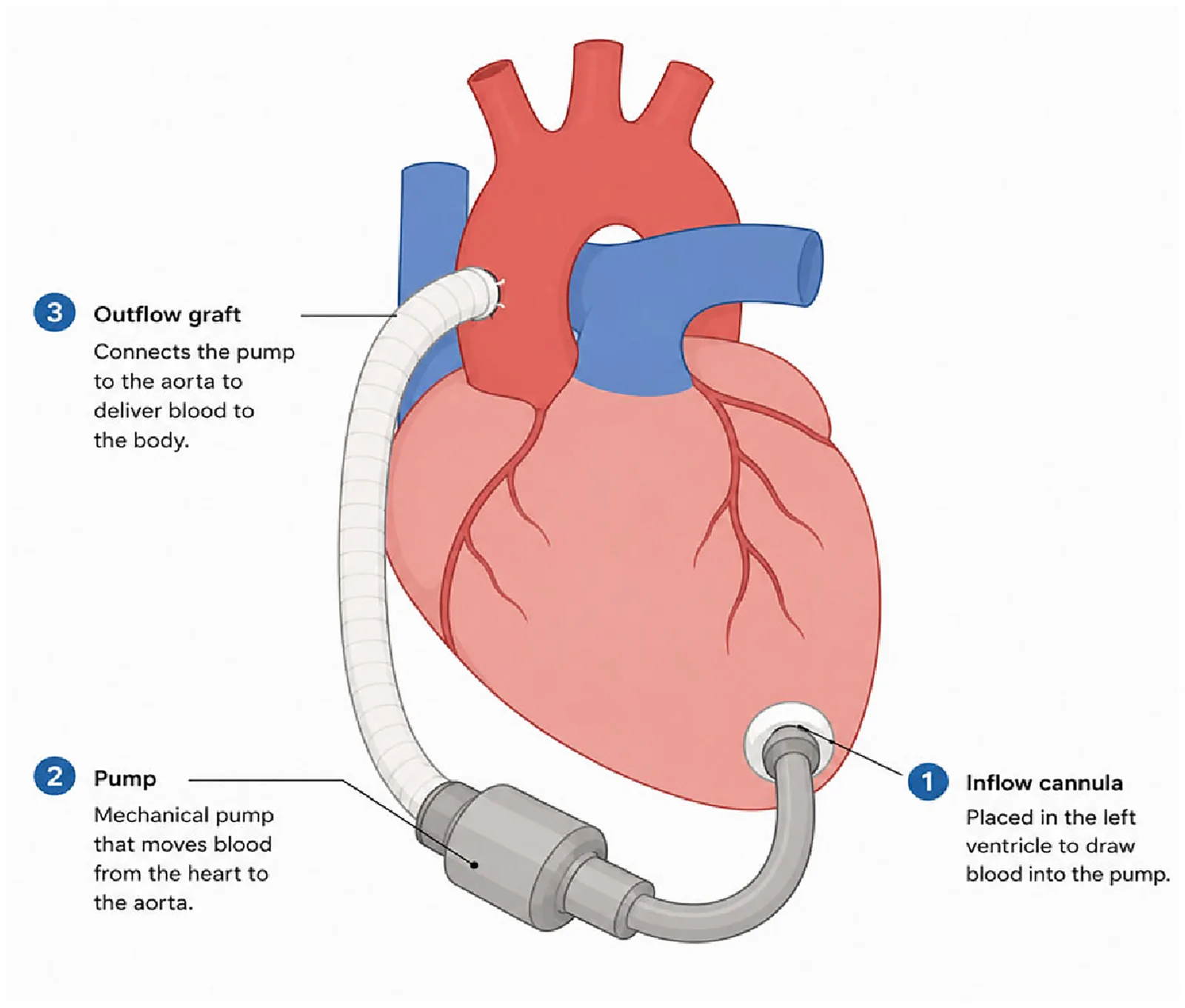
Simplified illustration of an implanted left ventricular assist device (LVAD) demonstrating the inflow cannula, the centrifugal pump, and the outflow graft anastomosed to the ascending aorta.

## Case report

A 71-year-old male with hypertensive cardiomyopathy and end-stage heart failure underwent implantation of a HeartMate 3 LVAD as destination therapy in June 2022; the procedure was performed in combination with biological aortic valve replacement and surgical closure of the left atrial appendage. The postoperative course was uncomplicated, and the patient remained clinically stable for more than 3 years with New York Heart Association class III functional status.

**Fig 2:**
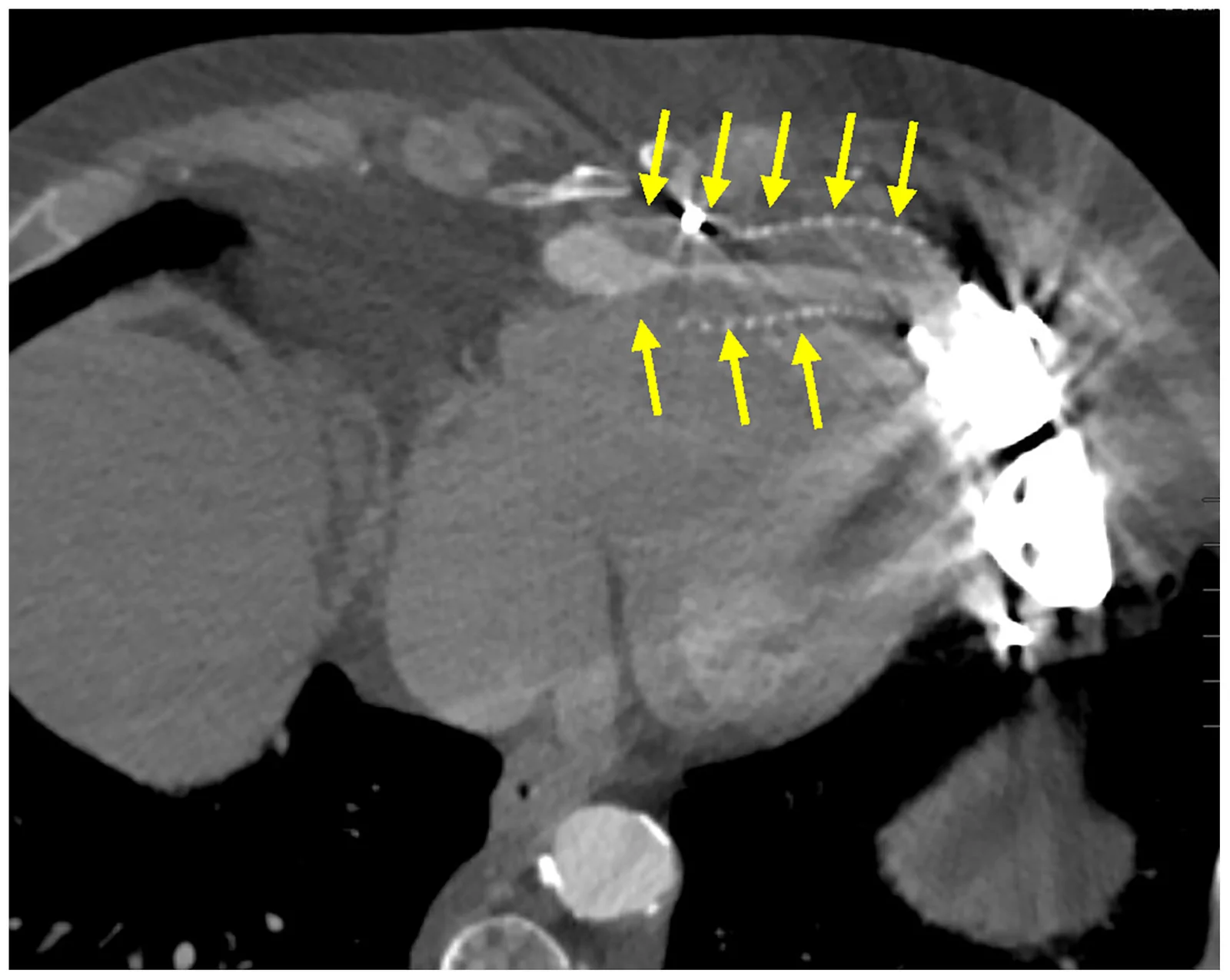
Preprocedural computed tomography angiography (CTA) demonstrating long-segment thrombosis and high-grade stenosis (*yellow arrows*) of the left ventricular assist device (LVAD) outflow graft within the bend-relief segment.

After experiencing a single self-limited low-flow LVAD alarm, the patient was admitted for evaluation. On presentation, the patient was hemodynamically stable with no signs of congestion or end-organ hypoperfusion, and his international normalized ratio (INR) was therapeutic at 2.1. CTA of the chest revealed a new long-segment, high-grade stenosis of the LVAD outflow graft. Despite intensified anticoagulation, repeat CTA showed progression of the lesion (Fig 2). Following multidisciplinary heart-team discussion, surgical revision was considered prohibitively high risk because it would have required redo sternotomy and complex cardiac reoperation in a patient with a previously implanted destination-therapy LVAD. Percutaneous endovascular intervention was therefore recommended.

**Fig 3:**
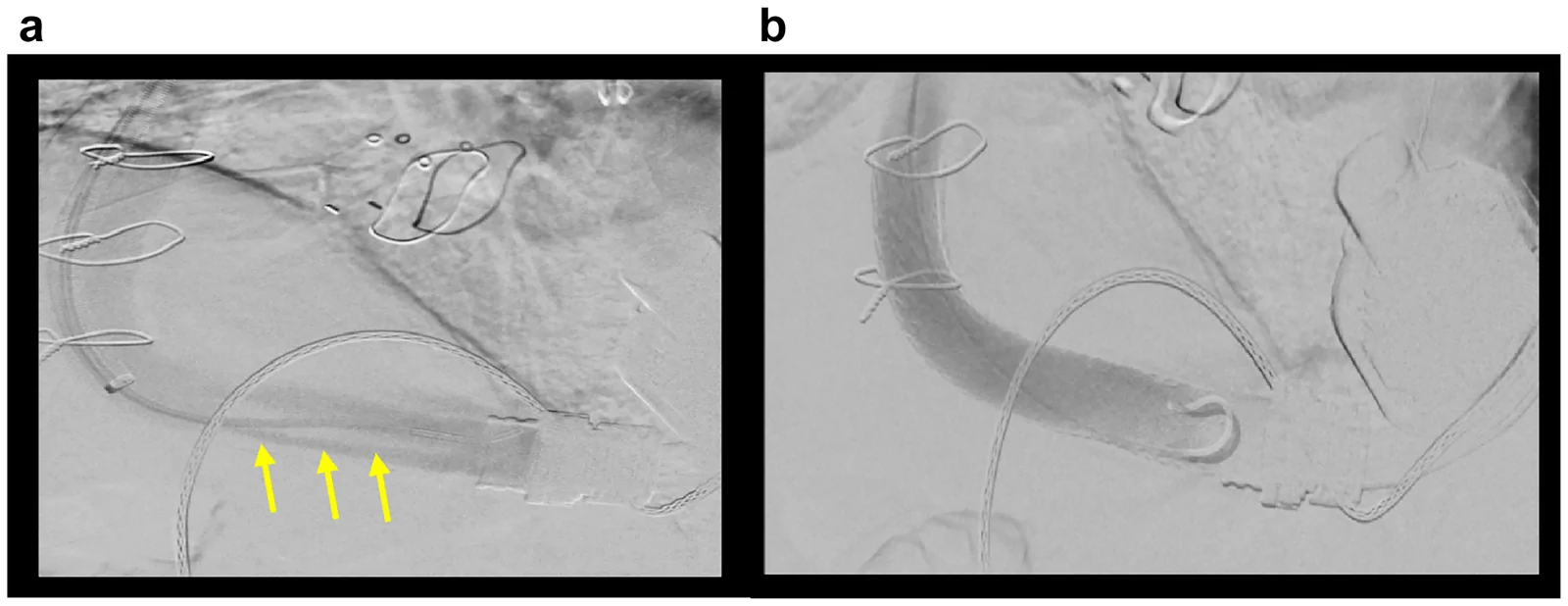
Angiography showing **(a)** critical outflow graft stenosis (*yellow arrows*) prior to intervention and **(b)** restoration of graft patency with unobstructed flow following stent implantation.

Under general anesthesia, right brachial arterial access was obtained, and intravenous heparin (5000 IU) was administered to maintain an activated clotting time above 250 seconds. Diagnostic aortography confirmed critical stenosis at the bend-relief segment of the outflow graft (Fig 3, *a*). The lesion was crossed with a guidewire, and a self-expanding nitinol venous stent (BeYond, 12 × 80 mm [Bentely InnoMed GmbH]) was positioned proximally near the pump anastomosis. To facilitate accurate deployment and minimize interaction with the pump impeller, the LVAD was briefly deactivated under invasive hemodynamic and transesophageal echocardiographic monitoring, with vasopressor support available.

After stent deployment, the guidewire was withdrawn into the stent lumen, and the LVAD was restarted. A second overlapping stent of identical size was deployed distally to fully cover the stenotic segment, followed by postdilatation with a 12 × 40 mm noncompliant balloon (Atlas Gold [BD/Bard Peripheral Vascular]). Final angiography demonstrated full expansion of both stents, restoration of graft patency, and brisk flow into the ascending aorta (Fig 3, *b*). The patient was extubated in the catheterization laboratory and transferred to the intensive care unit in stable condition, with immediate improvement in the LVAD flow from 2.8 to 3.7 L/min.

**Fig 4:**
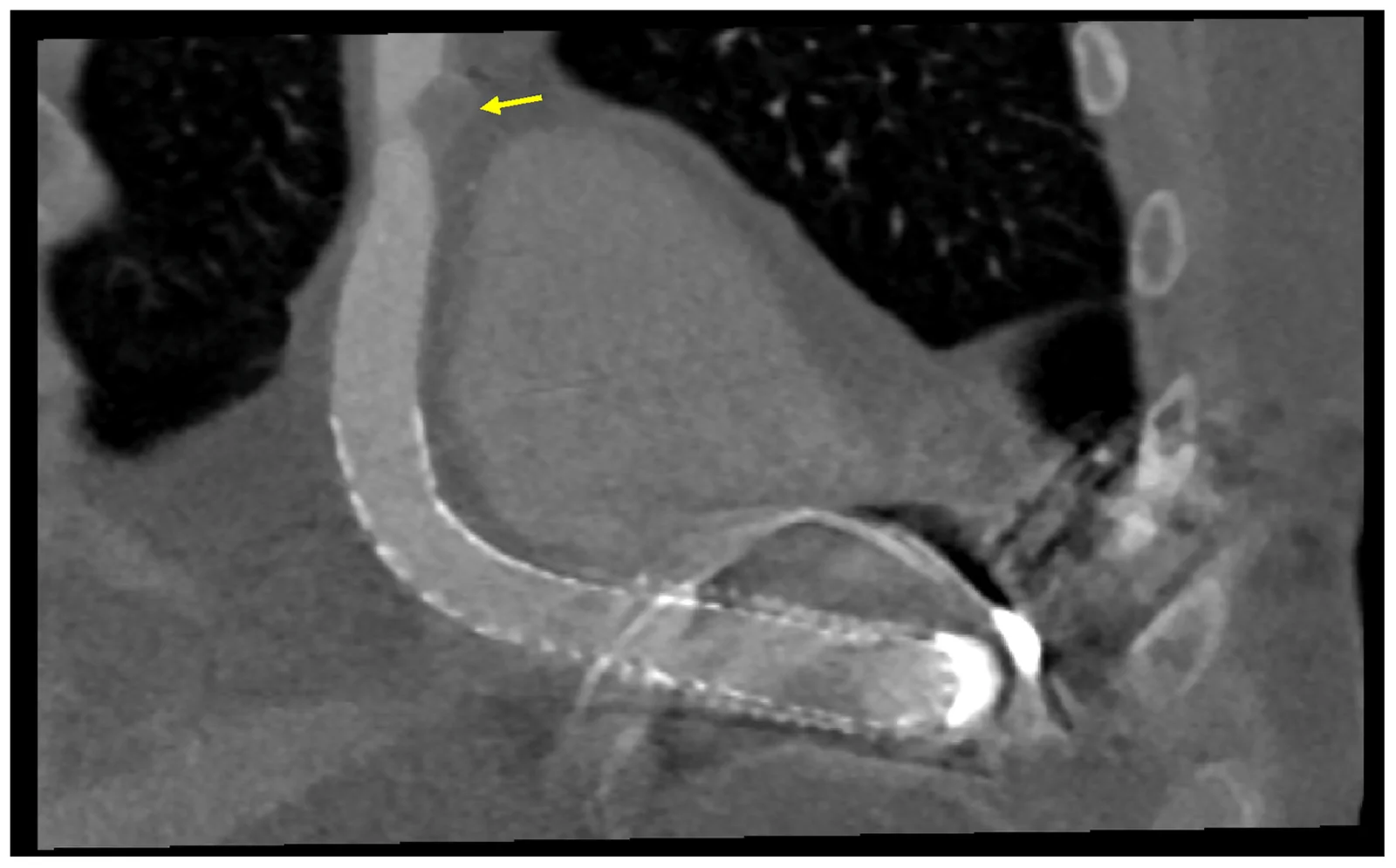
Follow-up computed tomography angiography (CTA) was obtained, showing patent proximal stents and a remaining short-segment thrombus (*yellow arrow*) distal to the stents at the anastomosis to the ascending aorta.

Routine follow-up CTA on postprocedural day 4 revealed a short distal stenosis consistent with thrombus beyond the stented segment near the ascending aortic anastomosis, while the proximal stents remained patent (Fig 4). The patient was asymptomatic, with stable hemodynamics and no further LVAD alarms. Given the embolic and neurologic risk associated with further graft manipulation near fresh thrombus, a conservative strategy was adopted with intensified anticoagulation (target INR, 2.5-3.0) and therapeutic heparinization, along with close imaging surveillance. The patient was discharged 8 days after the procedure with an INR of 2.7. Follow-up CTA performed 2 weeks after the intervention demonstrated persistent distal thrombotic stenosis without in-stent thrombosis, and continued imaging surveillance was recommended.

Written informed consent was obtained from the patient for publication of this case report and accompanying images.

## Discussion

Late OGO is an increasingly recognized complication across LVAD platforms and may be underdiagnosed because its often-subtle clinical presentation.^1^^,^^2^^,^^4^ In HeartMate II systems, OGO typically reflected intraluminal thrombotic or fibrotic disease with significant thrombus burden and higher embolic risk during intervention.^7^^,^^8^ In contrast, HeartMate 3-specific series have described late extrinsic compression of the outflow graft, particularly at the bend-relief segment, often without hemolysis or progressive device parameter deterioration.^3^^,^^4^

The present case exhibited features of both phenotypes, supporting the concept of a mixed or spectrum-based pathophysiology. CTA played a pivotal role in diagnosis, procedural planning, and postinterventional surveillance, reinforcing its status as the cornerstone imaging modality for suspected OGO.^3^^,^^4^ Laboratory markers and LVAD log-file analysis alone may be insufficient to exclude significant obstruction, particularly in HeartMate 3 devices.

When surgical revision is contraindicated, percutaneous endovascular stenting has been shown to rapidly restore device flow and improve hemodynamics.^5^^,^^6^^,^^9^^,^^10^ Technical strategies reported for HeartMate 3 interventions—including upper-extremity access and temporary LVAD pump speed reduction or brief deactivation during proximal deployment—were safely applied in this case without hemodynamic instability.^3^^,^^9^^,^^10^

Stent selection is a critical procedural consideration and should be individualized according to lesion morphology, thrombus burden, and graft geometry. In the present case, although thrombus was present, the lesion was predominantly characterized by long-segment narrowing within the curved bend-relief segment rather than extensive intraluminal thrombus requiring exclusion. Therefore, a flexible self-expanding nitinol venous stent was selected because its high conformability and sustained radial force allow scaffolding of the curved outflow graft while minimizing kinking. In contrast, balloon-expandable and self-expanding arterial stents may provide less conformability in this anatomical setting, whereas covered stents may be preferable in the presence of extensive thrombus or graft disruption but would increase construct rigidity, require advancement of a bulkier delivery system near the pump anastomosis, and offer no clear procedural advantage for this lesion morphology.^3^^,^^9^^,^^10^

Distal migration or propagation of obstructive material remains a recognized procedural risk and has been reported after stenting in both thrombotic HeartMate 2 cases and HeartMate 3 series.^3^^,^^7^^,^^8^ The optimal management of newly detected distal lesions remains controversial. In clinically stable patients, conservative management with intensified anticoagulation and close surveillance may represent a balanced strategy to mitigate embolic risk while avoiding potentially hazardous repeat intervention. In our patient, anticoagulation was intensified with therapeutic heparinization and an increased INR target of 2.5 to 3.0, balancing thrombus progression against the risk of repeat endovascular intervention.

## Conclusions

Percutaneous endovascular stent implantation offers an effective treatment option for late LVAD OGO in selected patients. However, the risk of distal migration of obstructive material and postintervention thrombosis underscores the necessity for meticulous procedural planning and individualized postintervention management strategies.

The authors thank the multidisciplinary heart team and catheterization laboratory staff for their contribution to patient care.

## Consent for publication

Written informed consent was obtained from the patient for publication of this case report and accompanying images.

## Data availability

All data generated or analyzed during this study are included in this published article.

## Funding

None.

## Disclosures

None.
